# P08-09 The promotion of walking through nudging. Evaluation of the app „Time2Walk“

**DOI:** 10.1093/eurpub/ckac095.122

**Published:** 2022-08-29

**Authors:** Sylvia Titze, Sowannry Em, Sandra Kniely, Verena Gumhold, Andreas Freidl, Maria Reiner, Mario Platzer, Thomas Wernbacher

**Affiliations:** 1 Institute of Sport Science, University of Graz, Graz, Austria; 2 Ovos GmbH, Wien, Austria; 3 Managerie, Graz, Austria; 4 Yverkehrsplanung, Graz, Austria; 5 Zentrum für Angewandte Spielforschung, Donau-Universität Krems, Krems, Austria

**Keywords:** walking, nudge, app

## Abstract

**Background:**

Walking is an appropriate mode to increase or maintain the physical activity behavior. However, physically inactive adults often need support in order to increase their physical activity level. The aim of the presentation is to inform about the nudging framework within the app Time2Walk, the recruitment of the study participants for both time points, and the development of the walking behavior over time.

**Methods:**

The study participants were recruited via social media, press releases, advertising material and personal contacts. A pre-post single group study design was applied using an online questionnaire before and after the intervention.

The intervention refers to a not so distant dystopian future where the city Graz (Austria) suffers from pollution, traffic chaos as well as climate change. Via regular walking as well as visiting places such as parks or other points of interest the users of the app were rewarded with tokens and could contribute to reversing the pollution. Four different types of behavioral nudges were applied during the use of Time2Walk in order to motivate people to walk more: awareness-raising (information about health and traffic related effects), social (information about guided walking tours), rewarding (token-based rewards), and stabilizing (daily walking targets) nudges.The app was developed as a central hub for conveying the nudges and for raising the players' awareness. By means of Wilcoxon-tests, changes in behavior categories were investigated.

**Results:**

Three hundred and forty seven people registered and 145 (42%) filled in the first questionnaire: 65% women, mean age 32 years (*SD* ±11) and 57% > high-school certificate. Of those, 31 (21%) filled in the the questionnaire a second time. Based on the stages of the Transtheoretical Model as well as on subjective assessment of the weekly walking behavior participants significantly raised their frequency of walking (20% increased walking from 4 or less days/week to ≥ 5 days/week).

**Conclusions:**

Different interdisciplinary expertise is essential for the development of the app-prototype including the nudging framework. Despite push-notifications, reminders through the newsletter and social media the response rate was low. In general, the nudging framework was successful in the increase of the perceived walking behavior.

